# Adult‐type granulosa cell tumor: An unusual testicular tumor

**DOI:** 10.1002/ccr3.5072

**Published:** 2021-11-09

**Authors:** Alain Mwamba Mukendi, Joelle Bukumbabu Mukendi, Ahsan Ahmad, Nompumelelo Mtshali

**Affiliations:** ^1^ Division of Urology Department of Surgery Faculty of Health Sciences Charlotte Maxeke Johannesburg Academic Hospital Johannesburg South Africa; ^2^ Faculté de Médecine Université de Kinshasa Kinshasa Democratic Republic of Congo; ^3^ Department of Paediatric Haematology and Oncology Nursing Services Chris Hani Baragwanath Academic Hospital Johannesburg South Africa; ^4^ Department of Anatomopathology Faculty of Health Sciences University of the Witwatersrand Johannesburg South Africa

**Keywords:** adult type, granulosa cell tumor, juvenile type, sex cord stromal tumor, testicular tumor

## Abstract

Adult type granulosa cell tumours are extremely rare. Albeit mostly benign, 10% have malignant potential associated with unfavorable pathological features. The present case with the longest history duration, shows that size alone may not necessarily be unfavorable prognosticator when not associated with other factors.

## Graphical abstract

Adult‐type granulosa cell tumors are extremely rare. Albeit mostly benign, 10% have malignant potential associated with unfavorable pathological features. The present case with the longest history duration shows that size alone may not necessarily be unfavorable prognosticator when not associated with other factors.

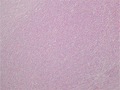

## 1 INTRODUCTION

Adult type testicular granulosa cell tumour is a very rare sex cord testicular tumour. We report the first case of this unusual testicular tumour with the longest history duration. Moreover, despite the presence of only one unfavorable prognostic factor, size greater than 5 cm, the tumour behavior was utterly benign.

Tumors arising from the testis comprise a diversity of neoplasms with wide‐ranging histopathological, biological behaviors, and clinical findings.^1^ Testicular granulosa cell tumors are extremely rare and can be classified into juvenile and adult types. The juvenile type is usually benign, whereas the adult type has a clinical behavior that is difficult to predict.^2^ Less than 100 cases of adult‐type granulosa cell tumors have been reported to date of which the youngest was 12 years old of age and none of them had over 30 years history duration with reported duration varying from 2 weeks to 10 years.^3^, ^4^, ^5^

## 2 CASE REPORT

A 49‐year‐old male known Diabetic mellitus type 1 and hypertensive both well controlled on treatment presented with a right testicular mass since childhood which he first noticed when he was about 12 years old. He reported that the mass had never been painful and started increasing in size in the last 2 years before his visit. He also experienced erectile dysfunction which he believed is Diabetes Mellitus related. Physical examination was essentially normal except for an enlarged and hard right testis measuring about 9 cm ×9 cm ×8 cm. No gynecomastia was noted. Tumor markers including beta‐HCG = <1 IU/L (normal range: <1 IU/L) and alpha‐feto protein = 5.0 μg/L (normal range: <10 μg/L) were done and were all within normal ranges except for lactate dehydrogenase (LD) =207 U/L (normal range: 48–115) which was elevated. Scrotal ultrasound showed a solid and heterogenous lesion with multiple cystic components. The patient underwent a right radical orchidectomy. CT abdomen and pelvis done in perioperative period and 2 years after surgery (CT) showed no signs of distant metastases, residual disease, or recurrence progression of disease.

**FIGURE 1:**
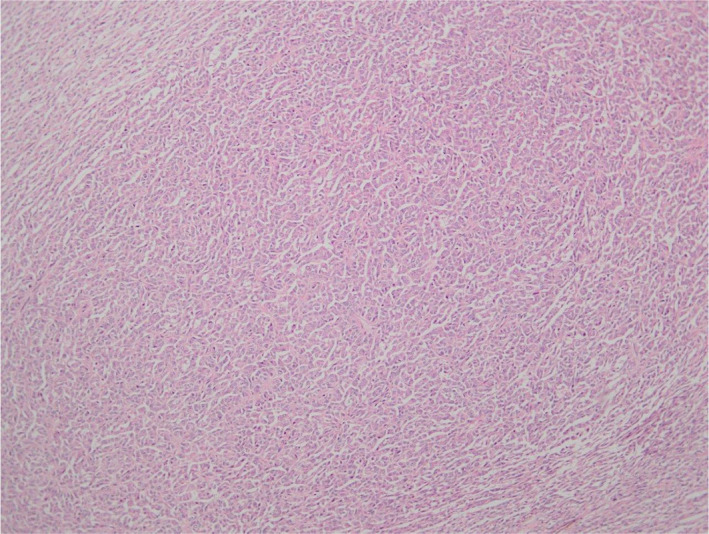
Sections of a well‐demarcated neoplasm with a variety of growth patterns including diffuse, cords and trabeculae, and Call‐Exner bodies

**FIGURE 2:**
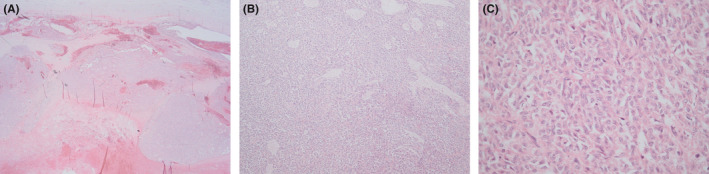
(A) Microfollicular and (B) macrofollicular growth patterns. The neoplastic cells have elongated nuclei and round to oval morphology. (C) Nuclear grooves are evident

**FIGURE 3:**
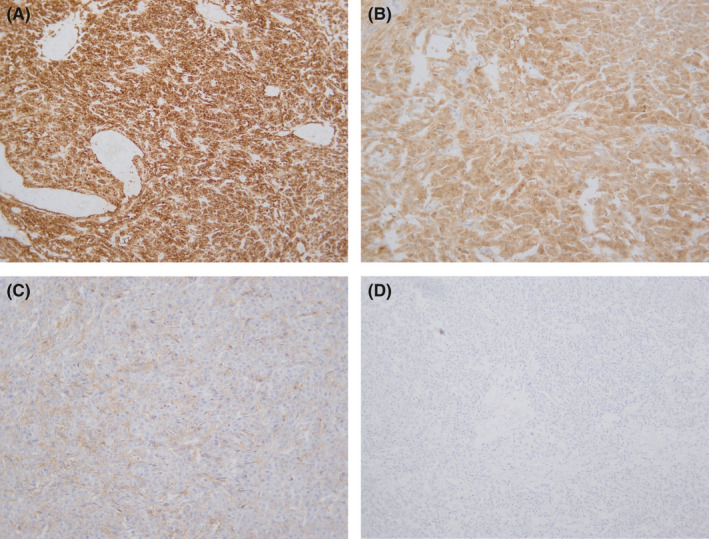
Immuno‐histochemical slides showing strong and diffuse positive staining with (A) Inhibin, (B) S100, and (C) CD99. (D) Negative staining with SALL4 is noted

On histopathologic analysis, the neoplasm had a maximum diameter of 85 mm. The tumor was well circumscribed. Histology showed sections of a well‐demarcated neoplasm with a variety of growth patterns including diffuse, cords, and trabeculae (Figure 1). Call‐Exner bodies were noted (Figure 1). Microfollicular and macrofollicular growth patterns were also observed (Figure 2A,B). The neoplastic cells had elongated nuclei and round to oval morphology. The cytoplasm was pale and scanty. Nuclear grooves were evident (Figure 2C). Mitotic activity was rare. No cellular atypia and no necrosis were observed. The tumorsurrounding testicular tissue showed no evidence of germ cell neoplasia in situ. No heterologous mucinous epithelium or hepatoid differentiation was seen. The immune‐histochemical workup showed strong and diffuse positive staining with Inhibin (Figure 3A), S100 (Figure 3B), and CD99 (Figure 3C). Negative staining with SALL4 was noted (Figure 3D).

## 3 DISCUSSION

Granulosa cell tumors are sex‐cord neoplasms of the gonads commonly arising in ovaries but are much rarer in men. They are classified into either juvenile or adult type. The juvenile type mostly occurs in the first 6 months of life and has a benign course, whereas the adult type even though mostly benign, 10% have malignant potential.^5^, ^6^ Adult‐type granulosa cell tumor usually manifests as a painless scrotal swelling; the minority of cases may present with gynecomastia.^2^, ^7^ Reported cases found in the literature presented between 2 weeks to 10 years of onset swelling. Our patient is the first report with over 30 years history duration.^3^, ^4^, ^5^

The youngest ever reported case of the adult type was 12 years old, whereas the oldest subject was 87 years old.^4^

The present case was 49 years old, and he reported to have had the swelling since he was 12 years old and it was never painful. None of the precedent case reports had such long history duration. No gynecomastia was observed on physical examination.

The definitive diagnosis of adult granulosa cell tumor is made by histopathologic examination, and it is mostly based on microscopic and morphologic features. A variety of microscopical growth patterns have been described including diffuse, insular, trabecular, solid, macrofollicular, microfollicular, gyriform, predominantly cystic, or pseudomembranous patterns. They may exist solely or in combination. Morphologically, granulosa cells are distinguished by their coffee‐bean like, angulated and centrally grooved nuclei. The presence of small spaces filled with hyalinized basement membrane material or eosinophilic fluid scattered between well‐differentiated granulosa cells, called Call‐Exner bodies, is pathognomonic. However, they are not always observed and therefore, are not essential to diagnosis. In a minor subset of cases, FOXL2 C123W gene mutation is present but it is less frequently observed in testicular granulosa cells tumors in comparison to its ovarian counterpart.^5^, ^6^, ^7^, ^8^ In the present case, the neoplasm was arranged in a combined trabecular, diffuse, corded, microfollicular, and macrofollicular patterns. Focal Call‐Exner bodies were identified. FOXL2 cytogenetic was not done as it is not indispensable to the diagnosis.

The malignant behavior of this rare entity is usually associated with unfavorable pathological features such as size >5 cm, presence of necrosis, >5 mitosis per 10 high power fields, marked nuclear atypia, lymphovascular invasion, extratesticular involvement, and gynecomastia. The most common sites of metastases include retroperitoneal lymph nodes, and the less common sites include liver, bones, and the lungs.^6^, ^8^, ^9^ Our patient only had one of these pathological features specifically the size greater than 5 cm. However, the process was completely benign in view of the history duration and the absence of metastases or evidence of disease recurrence or progression.

In terms of treatment, the initial management is radical orchiectomy or testis sparing surgery/wedge excision for smaller and/or benign tumors which in most cases are curative. Retroperitoneal lymph node dissection, platinum‐based chemotherapy (e.g., Bleomycin, Etoposide, and Cisplatin), and adjuvant radiotherapy for metastatic disease. In addition, there has been a report of an advanced case of testicular granulosa cell tumor that fractionally responded to Pazopanib, an angiogenesis inhibitor after initial resistance to cytotoxic chemotherapy.^5^, ^6^, ^8^ Radical orchidectomy was sufficient in our case as there was no evidence of local or distant metastases.

## 4 CONCLUSION

Adult‐type granulosa cell tumors are extremely rare. Albeit mostly benign, 10% have malignant potential associated with unfavorable pathological features. The present case shows that size alone may not necessarily be unfavorable prognosticator when not associated with other factors. It may therefore be important to correlate size with duration of mass, and a need for a ratio duration/size maybe essential in case series or consider a broad study with the aim to establish a prognostic score based on the presence or not of those pathological features correlated with disease progression or recurrence.

## CONFLICT OF INTEREST

The authors have declared that no conflict of interest exists.

## AUTHOR CONTRIBUTIONS

AMM conceived, designed the study, wrote the manuscript, revised it for critical content, and prepared the manuscript for submission. JBM wrote part of the manuscript. NM provided histopathological content and slides. AA revised the manuscript for critical content. They all approved the final version of the manuscript.

## CONSENT

Written informed consent was obtained from the patient for publication of this manuscript and accompanying pictures. A copy of the written consent is available for review by the Editor‐in‐Chief of this journal.

## Data Availability

The data that support the findings of this study are available from the corresponding author upon request.
